# Why not try harder to prove that automated external defibrillators save lives?

**DOI:** 10.1007/s12471-019-1248-z

**Published:** 2019-02-28

**Authors:** P. Calle, N. Mpotos

**Affiliations:** 1 Emergency Department, General Hospital Maria Middelares, Ghent, Belgium; 2 Faculty of Medicine and Health Sciences, University Hospital, Ghent, Belgium; 3 Emergency Department, General Hospital Saint Lucas, Ghent, Belgium

We do not feel comfortable with the article ‘Changes in automated external defibrillator use and survival after out-of-hospital cardiac arrest in the Nijmegen area’ by Nas et al., which appeared in the December issue of *Netherlands Heart Journal* [1].

From the title, one might assume that this is a population-based study. In the ‘Methods’ section, however, it is stated that only patients transported to a single hospital were included, implying that cases without a resuscitation attempt, victims with prehospital termination of resuscitation and patients transported to other hospitals were excluded. As the numbers of excluded patients are not given, it is unsound to compare the results of this study with those of any other study. The same remark on selection bias has to be made regarding the comparison between the study cohort and the historical controls. An indicator of important differences between both groups may be the numbers of included cases per month: 7.3 in the study cohort (349 over 48 months) versus 5.3 in the historical controls (180 over 34 months). Regarding this issue, the authors claim in ‘Limitations’ that the rates of in-field termination and hospital transportation have been stable over the years. However, the data they refer to are limited to the 34-month study period for the historical controls [2]. Furthermore, the comparison of the outcome data from 2008–2011 and 2013–2016 may be flawed by any factor evolving over time other than bystander cardiopulmonary resuscitation and the use of automated external defibrillators (AEDs).

Undoubtedly, our criticism of the results can only be avoided by a randomised clinical trial. However, this does not mean that prospectively registered data are of no value when searching for evidence of improved survival by AED use of lay rescuers. We believe that a close look at key elements in the resuscitation attempt of all individual surviving patients is very helpful [3]. Specifically, one needs to know how many of the survivors received AED shocks before the arrival of the emergency medical services (EMS) and how many minutes elapsed between the delivery of these shocks and EMS arrival. Indeed, the survival of a particular patient can only be attributed to an AED if that device delivered a shock. Moreover, one should be aware that in the survivor group survival might also have occurred without AED shocks being delivered by a lay rescuer. Quantification of the surplus value of the AED shocks in a particular patient will always be an estimate, but as a rule of thumb one may use a 10–12% decrease in survival rate for every minute of delay between the AED shock by the lay rescuer and EMS arrival [4]. As a final point, we insist on the importance of data on the global population in the recruitment area to allow any increase of survival rate in a particular subpopulation to be put into perspective.

Unfortunately, the authors have no data on time intervals regarding AED use. We hope that they will take into account our suggestions for their future registration and in the reports on the data on AED use by lay rescuers.
